# Genetic Diversity in Schizophrenia: Developmental Implications of Ultra-Rare, Protein-Truncating Mutations

**DOI:** 10.3390/genes15091214

**Published:** 2024-09-17

**Authors:** Jacob D. Clarin, Nadia N. Bouras, Wen-Jun Gao

**Affiliations:** Department of Neurobiology and Anatomy, Drexel University College of Medicine, Philadelphia, PA 19129, USA; jc4532@drexel.edu (J.D.C.); nnb37@drexel.edu (N.N.B.)

**Keywords:** schizophrenia, genome-wide association study, neurodevelopment, rare variant

## Abstract

The genetic basis of schizophrenia (SZ) remains elusive despite its characterization as a highly heritable disorder. This incomplete understanding has led to stagnation in therapeutics and treatment, leaving many suffering with insufficient relief from symptoms. However, recent large-cohort genome- and exome-wide association studies have provided insights into the underlying genetic machinery. The scale of these studies allows for the identification of ultra-rare mutations that confer substantial disease risk, guiding clinicians and researchers toward general classes of genes that are central to SZ etiology. One such large-scale collaboration effort by the Schizophrenia Exome Sequencing Meta-Analysis consortium identified ten, high-risk, ultra-rare, protein-truncating variants, providing the clearest picture to date of the dysfunctional gene products that substantially increase risk for SZ. While genetic studies of SZ provide valuable information regarding “what” genes are linked with the disorder, it is an open question as to “when” during brain development these genetic mutations impose deleterious effects. To shed light on this unresolved aspect of SZ etiology, we queried the BrainSpan developmental mRNA expression database for these ten high-risk genes and discovered three general expression trajectories throughout pre- and postnatal brain development. The elusiveness of SZ etiology, we infer, is not only borne out of the genetic heterogeneity across clinical cases, but also in our incomplete understanding of how genetic mutations perturb neurodevelopment during multiple critical periods. We contextualize this notion within the National Institute of Mental Health’s Research Domain Criteria framework and emphasize the utility of considering both genetic variables and developmental context in future studies.

## 1. A Genetic Basis for Schizophrenia

Schizophrenia (SZ) is a debilitating psychiatric disorder affecting roughly 1% of the world population [1]. The disorder is defined by three distinct symptom domains: positive, negative, and cognitive. While positive symptoms are associated with delusional thinking as well as auditory and/or visual hallucinations, negative symptoms typically arise prior to the first psychotic episode and include reduced motivation, flattened affect, poverty of speech, and social withdrawal [2]. Negative symptoms may be primary (i.e., intrinsic) or secondary in nature, arising as a consequence of other pathological factors including positive symptoms, antipsychotic drug side effects, or substance abuse [3]. The current pharmacological treatments for SZ include antipsychotic drugs, such as dopamine receptor D_2_ antagonists/partial agonists [4], but are only effective in treatment of positive symptoms, in a subset of patients [4,5]. The lack of reliable treatment options for negative symptoms is owed to the fact that their underlying neurobiological mechanisms are poorly understood and possibly divergent [6]. Negative symptoms are therefore typically more chronically disabling throughout the illness. In addition to these treatment gaps in dealing with negative symptoms, medications targeted to alleviate positive symptoms often come with side effects, such as movement disorders including tardive dyskinesia. In the cognitive symptom domain, patients experience impairments in working memory, attention, goal-directed behavior, and verbal fluency [7]. Thus, the treatment options available for SZ at present do not holistically treat all symptom domains, and these symptoms exist on a spectrum individualized for each patient with their own, distinct response profiles to the prevailing therapeutic approaches. Therefore, a primary objective of current SZ research is to define the underlying pathophysiological processes that give rise to the disorder, across all symptom domains, for all patients. Identifying the neurobiological underpinnings of SZ is essential to developing more effective therapies to improve disease outcomes for all patients.

One point of entry to characterize the biological processes implicated in SZ is through genetic studies. SZ is considered a highly heritable disorder and is both polygenic, wherein multiple genetic variants may lead to convergent disease phenotypes, and pleiotropic, wherein one gene variant precipitates towards multiple disease phenotypes. Understanding how alternations in gene expression and protein function give rise to cognitive and behavioral dysfunction is therefore a pressing mission among clinicians and scientists alike. A crucial component to resolving these unknowns is investigating when neurodevelopment disruption of gene function precipitates and predisposes an individual to SZ pathology. In pursuit of the identification of genetic loci associated with a particular disorder, the advent of genome- and exome-wide sequencing has proven invaluable, allowing for the detection of both common and rare genetic variants correlated with a disease of interest. 

Within the last decade, a handful of these genome- and exome-wide association studies have unearthed single-nucleotide polymorphisms (SNPs) in hundreds of distinct genetic loci linked to SZ. Granted, it is unlikely that all SZ variants confer the same level of risk, as SNPs may localize to introns, intergenic regions, or within exons that may shift the reading frame during mRNA translation, resulting in protein truncation. These genetic studies have, by and large, identified common allelic variants with relatively weak effect sizes as opposed to strong effect or causal variants [8]. A proposed hurdle in identifying variants of large effect is that they are particularly deleterious, and thus exceedingly rare as a result of natural selection forces [9]. To overcome this issue, a whole-exome study comprised of nearly 25,000 SZ cases and 100,000 controls was carried out by the Schizophrenia Exome Sequencing Meta-Analysis (SCHEMA) consortium. This collaboration involved the aggregation of exome sequencing data across numerous institutions and enabled adequately powered identification of ultra-rare coding variants (URVs) in a set of ten genes associated with substantially high risk for SZ. The URVs identified are nonsense mutations that result in premature stop codons during mRNA-to-protein translation, putatively leading to subsequent protein truncation. While it should be emphasized that these mutations are not causative, they exhibited odds ratios (ORs) ranging from 3 to 50 [10]. As one of the largest sequencing studies of any complex trait at the time of publication, it provides the most explicit link to date between protein dysfunction and SZ pathology (outlined in Table 1). It should be acknowledged that although this study overcame several technical barriers, it is nonetheless subject to potential biases, including its mostly European (73%) case sample. This caveat should be kept in mind when considering the results.

Indeed, while there have been hundreds of common variants that contribute milder risk for SZ, we focus on these ultra-rare variants for two primary reasons: Since there are no causative genetic mutations for SZ identified to date, these ultra-rare, high-risk mutations are the closest genotype–phenotype link currently known. Also, missense mutations or SNPs occurring in non-coding regions can have variable effects, including promoter interactions, epigenomic alterations, or implications on mRNA splicing. Therefore, until these mutations are probed in vitro or in animal models, their effects on gene expression or protein function are mostly speculative. Frameshift variants, on the other hand, most likely result in protein truncation, and ultimately a loss of function. We also acknowledge that consideration of only ten genes in a clearly polygenic disorder has its caveats, and that conclusions from this review ought not to be overgeneralized, which has been the pitfall of prior all-encompassing theories of SZ pathology (the dopamine or altered fetal neurodevelopment hypotheses, for example [76,77]) that have since been proven insufficient to wholistically explain clinical phenomena. With the discovery of these rare variants, the genetic architecture of SZ is becoming more resolved and appears to be subserved by both common and rare risk variants that interact with an environment throughout the lifespan [8]. Our review of the literature and open-access gene expression databases, however, suggests that SZ risk likely does not arise from the disruption of a singular neurobiological system or neurodevelopmental epoch.

The developmental hypothesis for SZ was originally proposed in 1986 and reasoned that while symptoms arise in early adulthood, genetic and environment threats disrupt perinatal neurodevelopment to predispose individuals for the disorder [77,78,79,80]. This initial conceptualization of SZ as a neurodevelopmental disorder was updated in 1999 to a “two-hit” model wherein impaired perinatal development first induces vulnerability but a second disruption during the adolescent critical period ultimately results in manifestation of the disorder [81]. The notion that SZ risk does not increase at only one point in development is supported by both preclinical and clinical studies. Notably, a hierarchical clustering approach showed that while one subset of SZ risk genes is highly expressed prenatally, with low expression postnatally, a second subset shows the opposite expression pattern [82]. To gain insight into whether SCHEMA risk genes display a similar pattern, we utilized the BrainSpan human developmental gene expression database (brainspan.org) [83]. This database includes postmortem brain samples from 42 male and female individuals aggregated from multiple institutions. Surprisingly, we discovered that while these genes undergo variable regulation of expression throughout pre- and postnatal development, there were commonalities in expression dynamics. Important to note is that Singh et al. presented developmental expression information for all 10 genes in their supplementary data but did not rigorously discuss the implications of these expression data, as this was not the primary focus of the manuscript [10]. Here, our analysis (method as described thoroughly in [84] and based on protocols from Singh et al. [10]) of the expression of these genes in the human brain across development reveals three general patterns, summarized as:

- High Prenatal, Low Postnatal (*TRIO, SETD1A, SP4*);
- High Prenatal, Adolescent Increase (*RB1CC1, XPO7, CUL1, HERC1*);
- Low Prenatal, High Postnatal (*GRIA3, GRIN2A, CACNA1G*).

These groupings are meant to serve as an illustration of the heterogeneity of expression patterns these high-risk genes may exhibit and additionally draw attention to commonalities in these patterns. They are not intended to make any claims based on statistical significance. 

Disruption of essential genetic programs at different stages of development may produce divergent symptom profiles in individuals with SZ. Of particular interest are those genes that display enhanced expression during the adolescence–adulthood transition, given that SZ symptoms typically emerge during late adolescence to early adulthood. To ascertain if there are indeed overlaps in terms of the functional consequences in perturbation of these genes, we review genetic knockdown and knockout studies that shed light on the potential consequences of these protein-truncating mutations. We additionally emphasize developmental expression data, allowing for an exploration of how genetic risk factors may contribute to alterations in neurodevelopmental gene expression programs. In line with the National Institute of Mental Health’s Research Domain Criteria (RDoC) initiative, we posit that part of the symptomatic heterogeneity in SZ can be owed to genetic diversity across patients, and that risk genes are recruited to support proper neurodevelopment at varying stages. These rare protein-truncating mutations provide a window through which to explore how genetic threats may arise at multiple timepoints throughout brain development. 

## 2. High Prenatal Expression (*TRIO, SETD1A, SP4*)

As discussed above, disruption of prenatal neurodevelopmental processes has long been a proposed mechanism for SZ. Preclinical genetic perturbation studies focused on SCHEMA genes with high prenatal expression report deficits in neurite outgrowth and branching, stem cell proliferation and differentiation, as well as synaptic abnormalities. Note that the odds ratios presented in Table 1 are representative of Class I mutations that correspond to both PTVs and high-risk missense variants [10].

### 2.1. TRIO

**Figure 1:**
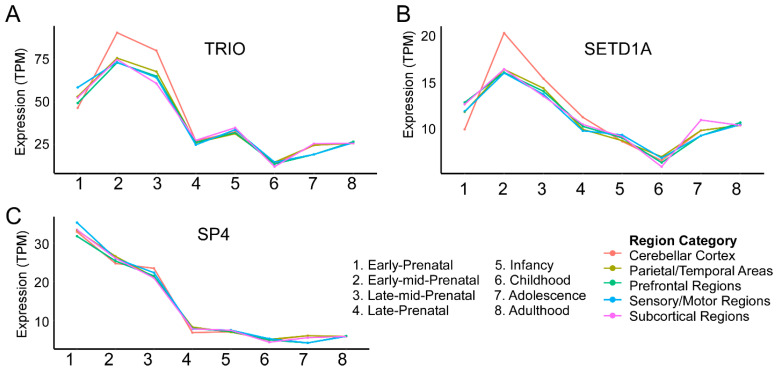
High-risk genes display high prenatal gene expression. mRNA expression from postmortem brain tissues across pre- and postnatal stages is quantified in units of transcripts per million (TPM). Of note is the relatively high expression during early development that gives way to a relative decrease into the postnatal period. Brain regions were placed into general categories as described in [84]. Expression timelines for *TRIO*, *SETD1A* and *SP4* are shown in (**A**), (**B**), and (**C**), respectively.

TRIO (Figure 1A), a guanine nucleotide exchange factor, is involved in actin cytoskeleton reorganization during neurodevelopment [11,12]. Specifically, TRIO was found to negatively regulate neurite outgrowth developmentally and AMPA-mediated transmission in neuronal cell culture models [13]. From an in vivo standpoint, a mouse model exhibiting *TRIO* haploinsufficiency targeted specifically to pyramidal neurons shows reduced dendritic arborization, suggesting that it may serve as a break signal to prevent premature maturation of excitatory synapses and dendrite morphogenesis. Physiologically, these mice also exhibit decreased miniature excitatory synaptic current (EPSC) frequency and impaired long-term potentiation (LTP), while behaviorally, anxiety-like behavior and social deficits are notable phenotypes [14]. These studies exemplify how disruption of cytoskeletal regulation may impair a range of structural and functional readouts of nervous system development.

### 2.2. SETD1A

Another SCHEMA risk gene, *SETD1A* (Figure 1B), supports proper neurodevelopment through epigenetic regulation. A histone (H3K4) methyltransferase, SETD1A is essential for neurodevelopment, aiding in the essential processes of neurogenesis. Its importance is evidenced by the observation that its deletion in mice is embryonic lethal—at around E7.5 [19]. Mechanistically, embryonic deletion of SETD1A leads to a global decrease in H3K4 methylation, a reduction in pluripotency gene expression, and an overall increase in G1 stage cells [19]. Additionally, SETD1A was found to inhibit differentiation into mature neurons, suggesting that this gene helps maintain a proliferative state by regulating expression involved in proliferation and stemness [17]. Beyond these early developmental deficits, a mouse model of a missense mutation observed in patients (R913C) displays faster migration of neurons to superficial cortical layers [20]. Structurally, a haploinsufficiency in vitro model comprised of both excitatory and inhibitory neurons exhibited increased dendritic arborization and heightened network bursting activity [21]. These additional findings suggest that SETD1A is involved in epigenetic regulation of genetic programs that help control the birth of neurons during the prenatal period and subsequent establishment of connectively, postnatally. The last SCHEMA gene exhibiting relatively high prenatal expression encodes the zinc finger transcription factor, SP4, that similarly regulates gene expression. 

### 2.3. SP4

SP4 (Figure 1C) regulates the expression of a number of genes to aid in diverse processes ranging from neuronal differentiation to dendritic maturation, as well as learning and memory [23]. Its binding to GC-box motifs upstream of gene promoters is generally associated with transcriptional activation [24]. Of note, SP4 has been shown to regulate the expression of NMDA and AMPA receptor subunits [25,26], the catalytic enzyme serine racemase, which is responsible for the production of the NMDA receptor co-agonist, D-Serine [27], and Neurotrophin-3, a growth factor involved in neuronal differentiation and synaptic development [28]. Interestingly, in silico analysis revealed that SZ risk genes exhibit a GC-box motif more significantly than would be expected by chance [29]. This indicates that *SP4* mutations may lead to broad disruption of several genetic programs that support neurodevelopmental processes that are altered in SZ. In terms of behavioral changes associated with *SP4* dysfunction, SP4 hypomorphic mice also show decreased performance in a hippocampus-dependent contextual memory task. The discovery of URVs in *SP4* and *SETD1A* supports current evidence that alterations in transcription factor activity and epigenetic processes increase risk for SZ [85]. 

## 3. High Prenatal Expression, Adolescent Increase (*RB1CC1*, *XPO7*, *CUL1*, *HERC1*)

This set of SCHEMA risk genes is compelling in terms of SZ pathology because of their relatively high expression during two relevant neurodevelopmental periods: early prenatal development and the transition from adolescence to adulthood. Adolescence and early adulthood are key developmental timepoints in SZ, as disease onset typically occurs between the ages of 16 and 30 [2]. To support the transition into adulthood, gene expression in the brain must be reorganized, and it has been documented that SZ risk genes, in particular, undergo dramatic changes in their expression trajectories during adolescence in early adulthood [86]. Also, worth noting about these genes is their shared increase in expression during infancy and subsequent expression drop-off during childhood. There are indeed genes that have established roles in both prenatal and postnatal neurodevelopment (such as the transcription factor *SOX6* [87,88]), suggesting that mutations in these genes could produce “two hits” along the neurodevelopmental timeline. Although the significance of these fluctuations in terms of neurodevelopment remains unknown, the following studies have established the involvement of these genes in multiple cellular processes including autophagy, dendritic pruning, and LTP induction. 

### 3.1. RB1CC1

**Figure 2:**
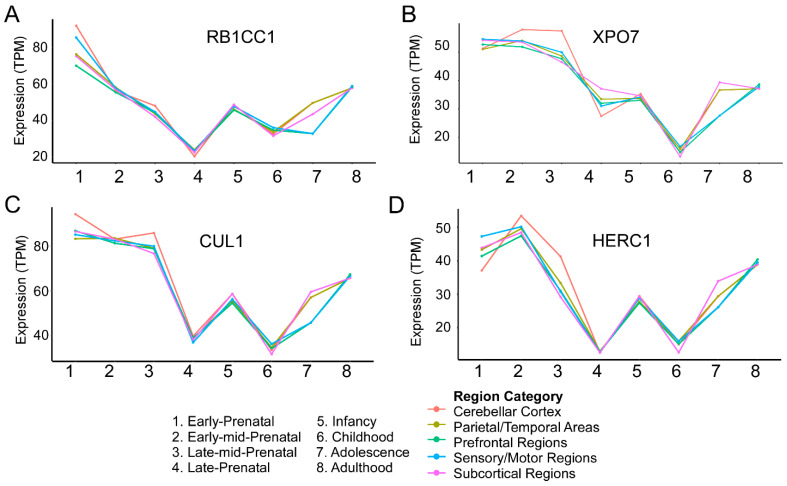
High-risk genes display high prenatal expression, followed by an increase in adolescence and adulthood. These SCHEMA risk genes exhibit multiple, prominent developmental expression changes that map onto previously implicated high-risk periods. mRNA expression is quantified in units of transcripts per million (TPM). Expression timelines for *RB1CC1*, *XPO7*, *CUL1*, and *HERC1* are shown in (**A**), (**B**), (**C**), and (**D**), respectively.

*RB1CC1* (Figure 2A) is a cytoplasmic protein that interacts with UNC-51-like kinases to induce autophagy in mammalian cells [32]. Unfortunately, there is a paucity of studies that have investigated the role of this gene specifically in the nervous system. However, the few existing reports are intriguing and indicate a crucial role in proper neurodevelopment. For example, deletion of this gene in a transgenic mouse model resulted in depletion and reduced differentiation of postnatal neural stem cells (NSCs), but not embryonic NSCs [33]. This finding aligns with the fact that *RB1CC1* is highly expressed both pre- and postnatally and could likely subserve different processes within each developmental context. Additionally, knockdown in neuronal cell culture limits neurite growth and promotes cell death [34], while a CRISPR-based knockout in cultured glutamatergic neurons exhibited autophagy deficiency as well as network electrophysiological hyperactivity, as assessed through micro-electrode arrays [35]. These results suggest that *RB1CC1* is a key regulator of autophagic processes in the maturing nervous system that, when disrupted, ultimately leads to morphological and neuronal excitability deficits. 

### 3.2. XPO7

Relatively little is known about *XPO7*, despite its high odds ratio (Figure 2B). *XPO7* encodes a nuclear exportin that is evidently involved in both the export and import of wide range of cargo proteins into the nucleus [37]. Considering its potential relevance to SZ, it is crucial to direct future studies to investigate how nuclear import and export subserved by XPO7 influences neurodevelopmental processes. 

### 3.3. CUL1

CUL1 (Figure 2C) is a core component of the SCF E3 ubiquitin ligase complex that is involved in ubiquitination of proteins targeted to the proteasome for degradation [38]. CUL1 is thought to be important for cell-cycle transitions during embryogenesis, and expectedly, homozygous mutant mice exhibit embryonic lethality at E6.5 [39]. These findings highlight the importance of the CUL1 specifically in neurons and in later developmental stages. RNAi experiments in cell culture resulted in apoptosis of dopamine neurons, specifically, suggesting that this protein promotes the survival of postmitotic neurons, perhaps in a cell-type-specific manner [40]. Another RNAi-based study in drosophila reported that knockdown resulted in impairments in dendritic pruning of sensory neurons in the *Drosophila melanogaster* peripheral nervous system [41]. More broadly, disruption of other components of Cullin1-based SCF E3 ubiquitin ligase complexes is reported to influence numerous neurodevelopmental processes such as neuronal morphogenesis, differentiation, migration, and presynaptic differentiation [42,43,44]. These findings reflect a general significance for CUL1 throughout prenatal and postnatal development, correlating with its human gene expression profile. 

### 3.4. HERC1

We next turn our focus to HERC1, a ubiquitin ligase also involved in tagging of proteins for proteasomal degradation. The role of HERC1 (Figure 2D) in nervous system function is relatively well studied, and mutations in the *HERC1* gene are associated with other pathologies, primarily intellectual disability [45,46,47]. The homozygous *HERC1* mutant mouse—termed *tambaleante,* or *tbl/tbl*—was first characterized as a model for cerebellar ataxia, but has since been utilized for interrogation of an expanded repertoire of neurological deficits [48]. As a caveat, *tbl* mice carry a missense mutation in the *HERC1* gene and show a marked increase of HERC1 protein expression in the brain [49]. This is indeed the most popular animal model for studying the function of HERC1 in the nervous system. Thus, the findings discussed here should be weighed with the consideration that a frameshift or other missense variants likely produce alternative phenotypic effects. Notably, *HERC1* mutant mice exhibit a number of presynaptic changes including decreased vesicle number, decreased number of docked vesicles, and a shorter active zone [50]. HERC1 thus regulates vesicle dynamics, and therefore, its dysfunction likely results in neural activity deficits. *Tbl* mice also exhibit disease-relevant neurological phenotypes, including associative fear learning deficits, as assessed by the passive avoidance test. These deficits were evident in both the short-term and long-term following learning [51]. Fear learning deficits in these mice are likely driven by neuroplasticity impairments, as LTP could not be induced in the lateral amygdala (LA), and there was a relative decrease in the amount of mature dendritic spines within this region [51]. These mutants also perform poorly in hippocampal-dependent learning paradigms, but surprisingly, LTP was unperturbed in Schaffer collateral–CA1 synapses, unlike in the LA [52]. While these studies highlight the importance of postnatal *HERC1* expression, there is a lack of mechanistic, early developmental models examining the impacts of prenatal *HERC1* disruption. These studies could provide more context for the *HERC1*-related phenotypes that emerge postnatally. 

## 4. Low Prenatal Expression, High Postnatal Expression (*CACNA1G, GRIN2A, GRIA3*)

The last SCHEMA group may be distinguished from those previously discussed by their relatively low prenatal expression. The two gene sets indeed exhibit expression spikes during infancy and adolescence, denoting their potential importance for the regulation of postnatal neurodevelopmental processes. Knockdown and knockout studies centered on these genes advocate for their importance in neuroplasticity and their relevance to SZ pathology. 

### 4.1. CACNA1G

**Figure 3:**
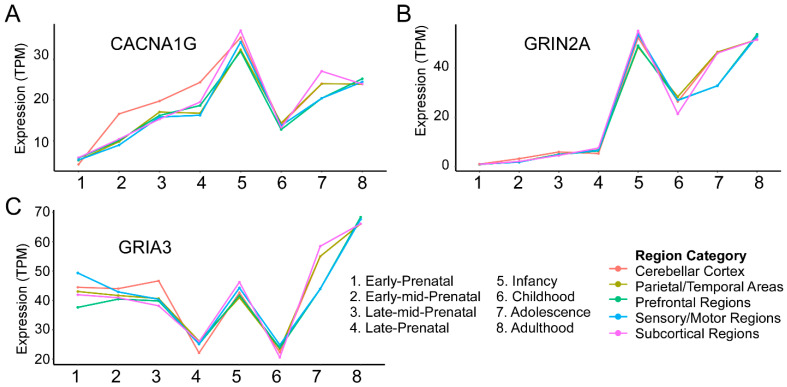
High-risk genes display relatively low prenatal gene expression and an increase in expression postnatally. The genes exhibit low or moderate relative gene expression during the prenatal period and a subsequent spike at multiple points throughout the postnatal period, including infancy, adolescence, and adulthood. mRNA expression is quantified in units of transcripts per million (TPM). Expression timelines for *CACNA1G*, *GRIN2A*, and *GRIA3* are shown in (**A**), (**B**), and (**C**), respectively.

*CACNA1G* (Figure 3A) encodes a T-type low-voltage-activated Ca_V_3.1 channel involved in the generation of low-threshold spikes and thus contributes to neuronal excitability as well as synaptic plasticity [53,54]. Case reports document an onset of epileptic encephalopathy during infancy patients with *CACNA1G* missense mutations [55]. The emergence of this epilepsy phenotype during infancy is curious given that *CACNA1G* expression in the brain peaks during this stage (Figure 3). Preclinically, a recent study utilizing thalamocortical assembloids to model *CACNA1G* knockout reported increased spontaneous firing activity and an overall increase in thalamocortical projections, reported as a consequence of increased axon growth cone migration [56]. This study is a compelling example of how one gene may affect neuronal activity and the establishment of functional circuitry. 

### 4.2. GRIN2A 

*GRIN2A* (Figure 3B) encodes the NR2A NMDA glutamate receptor subunit and has been studied extensively in the contexts of neural circuit maturation and neuroplasticity processes, including LTP. NMDA receptor hypofunction has received much attention within recent years as a prevailing mechanism for the pathophysiological of SZ [59]. Developmentally, there is a functional switch in NMDAR subunit composition early postnatally wherein expression of the NR2B subunit is gradually replaced by the NR2A subunit in many brain regions. This defines a critical period in which NMDAR kinetics transition from slow-decaying EPSCs to faster excitatory events, mediated by NR2A [60]. One exception to the timing of this early critical period lies in the prefrontal cortex, where this subunit switch was observed during adolescence and early adulthood [61]. These findings align with the BrainSpan expression data (Figure 3B), wherein *GRIN2A* expression increases dramatically during infancy, and then again into adulthood. Functionally, improper NMDA functional switching during circuit maturation can have severe consequences, as NR2A mutations may lead to the dominance of NMDA receptors composed of NR2B, that have more prolonged cation conductance, which may lead to neuronal hyperexcitability [62]. Cementing the role of *GRIN2A* in SZ pathology on a transcriptional level, a recent single-cell RNA-sequencing study provided evidence that *GRIN2A* mRNA expression was decreased in layer 5/6 neurons in the prefrontal cortex in postmortem SZ brains [63]. The repeated discovery of *GRIN2A* mutations across SZ clinical studies emphasizes the importance of NMDA receptor signaling within the context of the disorder [64].

### 4.3. GRIA3

*GRIA3* (Figure 3C) has previously been implicated in neurodevelopmental disorders [66,67]. It encodes for subunit 3 of the ionotropic AMPA receptor (GluA3) and is thus intimately linked to glutamatergic signaling in neurons. AMPA receptors are composed of four interchangeable subunits wherein two like subunits form a dimer and join with a second pairing of like subunits, forming a so-called “dimer of dimers” [68]. AMPA receptor subunit structure contributes to its function—GluA3-containing AMPA receptors are calcium-permeable, and, until recently, their roles in synaptic plasticity and affective behavior were understudied compared to their GluA1 and GluA2 counterparts [69,70]. A landmark study demonstrated that while GluA3-containing AMPA receptors in hippocampal CA1 synapses display low or no cation conductance under baseline conditions, increases in intracellular cAMP induce a high permeability state. Further experiments elucidated that β-adrenergic signaling can contribute to this rise in intracellular cAMP, serving as a neuromodulatory stimulus that triggers synaptic potentiation in GluA3-containing AMPA receptors [70]. Along these lines, mutations in *GRIA3* have been linked to decreased miniature excitatory postsynaptic current (EPSC) frequency and reduced amplitude of large EPSCs [71]. *GRIA3* knockout models in mice also display intriguing behavioral deficits, exhibiting increased sociability and aggressive phenotypes in male animals [72,73]. Interestingly, this aggressive behavior phenotype is recapitulated in patients with rare *GRIA3* mutations [73]. These findings coincide with a preponderance of evidence associating SZ with aggressive behavior. However, it should be emphasized that this does not imply that this population is on the whole more aggressive [74]. The high-risk URVs discovered in this gene and GRIN2A suggest that perturbation of neurotransmission and synaptic plasticity is a driving component of SZ pathology.

## 5. Preclinical Data Relevance to SZ Clinical Biomarkers

While the preclinical evidence presented support a role for SCHEMA risk genes across the spectrum of neurobiological function, it is important to discuss how these findings may relate to established clinical features of SZ. Notably, these genes appear to be pleiotropic, as they have been associated with additional neurological comorbidities (shown in Table 1). There are some key clinical features of SZ that may be relevant to the preclinical data presented. The first is a change in neural network activity, characterized by hyperactivity (as observed in models of *GRIN2A*, *CACNA1G*, *RB1CC1*, and *SETD1A*), particularly in the hippocampus [89] and amygdala [90]. This pathological shift in network excitability is likely a consequence of changes in synaptic excitation/inhibition balance, a proposed mechanism underlying SZ that is supported not only by the discovery of numerous synaptic genes in GWAS but also by abnormalities in cortical inhibitory interneurons observed in the brains of SZ patients post-mortem [91]. Likely related to dysregulation of excitation/inhibition balance are dendritic abnormalities (as observed in models of *CUL1*, *TRIO*, *SETD1A*, and *SP4*). Reductions in dendritic spine density, particularly in the prefrontal cortex, is a core pathological feature of SZ [92], highlighting the significance of investigating how arborization, spine formation, and pruning are differentially regulated in pathological states. Functionally speaking, learning and memory deficits (as observed in models of *SP4* and *HERC1*) are also found in SZ [93,94,95]. In terms of early development, most of the prenatal insults documented thus far are related to environmental events such as maternal infections or malnutrition [96]. Since many genes associated with SZ, including several presented here, have established roles in early neurodevelopmental processes, future studies should interrogate the potential influence of genetics upon neurological defects observed prenatally and early postnatally and how they may predispose an individual for developing SZ later in life. The relationships between preclinical and concrete biomarkers across these genes and others linked to SZ remain tenuous and require follow-up studies investigating the connection between high-risk mutations and disease-relevant neurological biomarkers. 

## 6. Genetic and Developmental Diversity Giving Rise to SZ Symptom Heterogeneity—Consideration of the NIMH RDoC Framework

**Figure 4:**
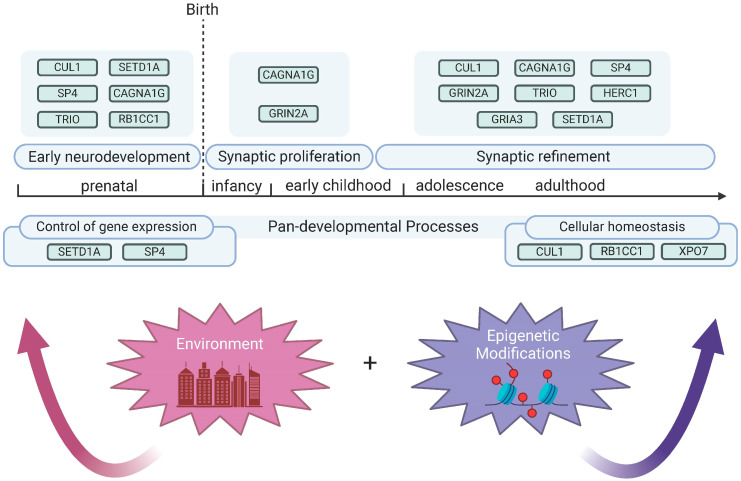
An updated neurodevelopmental risk model for schizophrenia. While some mutations may primarily impact pre- or postnatal developmental processes, others may bear influence across multiple developmental stages. The SCHEMA risk genes support early neurodevelopmental and circuit maturational events that are further impacted by environmental and epigenetic factors. Taken together, we postulate that the variable inheritance of different risk variants among SZ patients, and the differential impacts they may harbor across neurodevelopment, contribute to the heterogeneous nature of this disease population.

Despite years of careful experimentation, advocacy, and support from public and private research dollars, the underlying neurobiological mechanisms that drive all aspects of SZ pathology remain elusive. Part of the problems with a “unified hypothesis” stems from the heterogeneity in symptom presentation across clinical cases. Although a core set of symptom criteria is met, the severity of these symptoms is highly variable. This symptom heterogeneity additionally makes modeling SZ exceedingly difficult, and there is a dire need to employ new research paradigms to identify and characterize the fundamental perturbations that increase risk for development of the disorder. The RDoC initiative currently aims to adjust the approach of how psychopathologies are studied, instead placing an emphasis on factors that result in deviations from normal neurological function. In this framework, symptoms are distilled down to basic domains, including arousal, positive and negative valence, sensorimotor processing, as well as cognitive and social function. In a cross-sectional approach, pathological variations across these domains may be studied as a nexus of genetic, developmental, and environmental factors. Indeed, studies support that grouping clinical cases by neurobiological biomarkers can more effectively delineate clinical populations than typical diagnostic criteria, suggesting that these diagnostic criteria may sometimes fail to perfectly map onto common underlying neurobiological deficits [97]. Our review of the URVs, for example, suggests that there exists an interplay between genetic factors and critical brain maturational processes throughout development (Figure 4).

Protein-truncating variants identified in the SCHEMA study strongly suggest that future investigation of SZ risk genes should pay meticulous care in the selection of the variant being modeled, the developmental stage being studied, and which neurobiological systems are hypothesized to be affected. Given the evidence presented here as well as BrainSpan expression data, it is interesting to consider that certain gene variants may impose risk at more than one stage of development due to their recruitment in multiple genetic programs throughout the lifespan. Crucially worth noting, but not discussed here, is that biological sex, as well as environmental and epigenetic factors (outlined in Box 1), have an established role in SZ pathology [98] and thus add heightened complexity to an already unresolved disease etiology. Despite this complexity, the toolbox for probing the structure and function of the nervous system has never been more equipped to deal with complex diseases like SZ. As more high-powered clinical data come to the fore, it is the responsibility of researchers to adopt these novel strategies to best interrogate the influence of multiple risk factors to identify common disease mechanisms, and ultimately, therapeutic interventions (discussed in Box 2).

Box 1 **Considering Sex-Specific, Environmental, and Epigenetic Factors**

While this review highlights ultra-rare, high-risk mutations, it is essential to acknowledge the complex interplay of genetic, environmental, epigenetic, and sex-specific factors in SZ etiology. Environmental influences, including prenatal (malnutrition, maternal stress, infections during pregnancy, obstetric complications, and hypoxia during birth) and postnatal (urbanicity, season of birth, adverse childhood experiences, cannabis use, and social adversity) insults, contribute to disease risk (for a comprehensive review of environmental influences in SZ, see [99]). In addition, it is well established that epigenetic modifications, such as DNA methylation, histone modifications, and non-coding RNAs (reviewed in [100]), also play a role in disease progression. Lastly, sex differences, including higher prevalence and diagnoses in males, differential symptom onset and presentation, and sex-specific differences in neuroanatomy (reviewed in [101,102]), must be considered. Thus, given the complexity and heterogeneity of SZ, preclinical models must begin to integrate multifactorial designs to further elucidate the neurobiological mechanisms of SZ.

Box 2 **Outstanding Questions and Future Approaches**

Relevant questions:

- How do we wholistically study and model the impact of genetic risk factors in combination with environmental and epigenetic risk factors?
- How might genetic perturbation at varying neurodevelopmental timepoints be related to the symptomatic heterogeneity observed in psychiatric disorders like SZ?

Potential approaches:

- Experimentally, recent advances in CRISPR-Cas gene editing platforms may be applied in preclinical models to interrogate the effects of gene disruption at the developmental timepoint of the investigator’s choosing.
  ○ What may be the pre- vs. postnatal effects of gene knockout?
- Polygenic risk scores offer an avenue for detection of high-risk individuals.
  ○ First introduced in 2009 by the International Schizophrenia Consortium [103] and integrates genetic risk information from GWAS.
  ○ Polygenic risk scores are associated with a higher rate of conversion to psychosis from a high-risk state and significantly risk prediction in psychosis risk models [104,105].
  ○ Large-cohort studies such as SCHEMA allow for integration of rare variants into models dominated by common variants, potentially allowing for more accurate risk prediction.
  ○ Despite its advantages, there are additional ethical considerations that should be taken into account (as reviewed in [106,107]).

## Figures and Tables

**Table 1:** Overview of genes for which protein-truncating variants were identified by the SCHEMA consortium.

Gene	Function	Odds Ratio (OR) [10]	Selected Publications	Additional Neurological Phenotypes
TRIO	Guanine nucleotide exchange factor	5.02	[11,12,13,14]	Intellectual disability [15] Autism Spectrum Disorders [16]
SETD1A	Histone methyltransferase	10.3	[17,18,19,20,21]	Intellectual disability and developmental delay [22]
SP4	Transcription Factor	7.59	[23,24,25,26,27,28,29]	Bipolar disorder [30,31]
RB1CC1	Cytoplasmic protein involved in autophagosome assembly	10.0	[32,33,34,35]	Intellectual disability [36]
XPO7	Nuclear exportin	28.1	[37]	None yet reported
CUL1	SCF E3 ubiquitin–protein ligase component	44.2	[38,39,40,41,42,43,44]	None yet reported
HERC1	E3 ubiquitin–protein ligase	3.51	[45,46,47,48,49,50,51,52]	Intellectual disability [45,46,47]
CACNA1G	T-type low-voltage-activated calcium channel	4.25	[53,54,55,56]	Epilepsy [55] Cerebellar Ataxia [57] Cognitive impairment [58]
GRIN2A	NMDA receptor subunit	24.1	[59,60,61,62,63,64]	Intellectual disability and developmental delay [65] Epilepsy [65]
GRIA3	AMPA receptor subunit	20.1	[66,67,68,69,70,71,72,73,74]	Aggressive behavior [73] Developmental delay [75] Cognitive impairment [75] Epilepsy [75]

## Data Availability

The data presented in this study are available in the BrainSpan Atlas for the Developing Human Brain, publicly accessible at brainspan.org, referenced below [108].

## Abbreviations

EPSC, Excitatory postsynaptic current; H3K4, Histone H3 Lysine 4; LA, Lateral amygdala; Long-term potentiation, LTP; NSC, Neural stem cell; OR, Odds ratio; SCHEMA, Schizophrenia Exome Sequencing Meta-Analysis consortium; SZ, Schizophrenia; SNP, Single-nucleotide polymorphism; TPM, Transcripts per million; URV, Ultra-rare coding variant

## References

[1] Saha S., Chant D., Welham J., McGrath J. (2005). A systematic review of the prevalence of schizophrenia. PLoS Med..

[2] Owen M.J., Sawa A., Mortensen P.B. (2016). Schizophrenia. Lancet.

[3] Mosolov S.N., Yaltonskaya P.A. (2021). Primary and Secondary Negative Symptoms in Schizophrenia. Front. Psychiatry.

[4] Correll C.U., Schooler N.R. (2020). Negative Symptoms in Schizophrenia: A Review and Clinical Guide for Recognition, Assessment, and Treatment. Neuropsychiatr. Dis. Treat..

[5] Glatt S.J., Faraone S.V., Tsuang M.T. (2019). Schizophrenia.

[6] Millan M.J., Fone K., Steckler T., Horan W.P. (2014). Negative symptoms of schizophrenia: Clinical characteristics, pathophysiological substrates, experimental models and prospects for improved treatment. Eur. Neuropsychopharmacol..

[7] Bowie C.R., Harvey P.D. (2006). Cognitive deficits and functional outcome in schizophrenia. Neuropsychiatr. Dis. Treat..

[8] Sullivan P.F., Yao S., Hjerling-Leffler J. (2024). Schizophrenia genomics: Genetic complexity and functional insights. Nat. Rev. Neurosci..

[9] Zuk O., Schaffner S.F., Samocha K., Do R., Hechter E., Kathiresan S., Daly M.J., Neale B.M., Sunyaev S.R., Lander E.S. (2014). Searching for missing heritability: Designing rare variant association studies. Proc. Natl. Acad. Sci. USA.

[10] Singh T., Poterba T., Curtis D., Akil H., Al Eissa M., Barchas J.D., Bass N., Bigdeli T.B., Breen G., Bromet E.J. (2022). Rare coding variants in ten genes confer substantial risk for schizophrenia. Nature.

[11] Wang S., Bleeck A., Nadif Kasri N., Kleefstra T., van Rhijn J.-R., Schubert D. (2021). SETD1A Mediated H3K4 Methylation and Its Role in Neurodevelopmental and Neuropsychiatric Disorders. Front. Mol. Neurosci..

[12] Bellanger J.M., Astier C., Sardet C., Ohta Y., Stossel T.P., Debant A. (2000). The Rac1- and RhoG-specific GEF domain of Trio targets filamin to remodel cytoskeletal actin. Nat. Cell Biol..

[13] Seipel K., Medley Q.G., Kedersha N.L., Zhang X.A., O’Brien S.P., Serra-Pages C., Hemler M.E., Streuli M. (1999). Trio amino-terminal guanine nucleotide exchange factor domain expression promotes actin cytoskeleton reorganization, cell migration and anchorage-independent cell growth. J. Cell Sci..

[14] Ba W., Yan Y., Reijnders M.R., Schuurs-Hoeijmakers J.H., Feenstra I., Bongers E.M., Bosch D.G., De Leeuw N., Pfundt R., Gilissen C. (2016). TRIO loss of function is associated with mild intellectual disability and affects dendritic branching and synapse function. Hum. Mol. Genet..

[15] Katrancha S.M., Shaw J.E., Zhao A.Y., Myers S.A., Cocco A.R., Jeng A.T., Zhu M., Pittenger C., Greer C.A., Carr S.A. (2019). Trio Haploinsufficiency Causes Neurodevelopmental Disease-Associated Deficits. Cell Rep..

[16] Pengelly R.J., Greville-Heygate S., Schmidt S., Seaby E.G., Jabalameli M.R., Mehta S.G., Parker M.J., Goudie D., Fagotto-Kaufmann C., Mercer C. (2016). Mutations specific to the Rac-GEF domain of TRIO cause intellectual disability and microcephaly. J. Med. Genet..

[17] Sadybekov A., Tian C., Arnesano C., Katritch V., Herring B.E. (2017). An autism spectrum disorder-related de novo mutation hotspot discovered in the GEF1 domain of Trio. Nat. Commun..

[18] Mukai J., Cannavo E., Crabtree G.W., Sun Z., Diamantopoulou A., Thakur P., Chang C.Y., Cai Y., Lomvardas S., Takata A. (2019). Recapitulation and Reversal of Schizophrenia-Related Phenotypes in Setd1a-Deficient Mice. Neuron.

[19] Bledau A.S., Schmidt K., Neumann K., Hill U., Ciotta G., Gupta A., Torres D.C., Fu J., Kranz A., Stewart A.F. (2014). The H3K4 methyltransferase Setd1a is first required at the epiblast stage, whereas Setd1b becomes essential after gastrulation. Development.

[20] Yu X., Yang L., Li J., Li W., Li D., Wang R., Wu K., Chen W., Zhang Y., Qiu Z. (2019). De Novo and Inherited SETD1A Variants in Early-onset Epilepsy. Neurosci. Bull..

[21] Wang S., Rhijn J.V., Akkouh I., Kogo N., Maas N., Bleeck A., Ortiz I.S., Lewerissa E., Wu K.M., Schoenmaker C. (2022). Loss-of-function variants in the schizophrenia risk gene SETD1A alter neuronal network activity in human neurons through the cAMP/PKA pathway. Cell Rep..

[22] Kummeling J., Stremmelaar D.E., Raun N., Reijnders M.R.F., Willemsen M.H., Ruiterkamp-Versteeg M., Schepens M., Man C.C.O., Gilissen C., Cho M.T. (2021). Characterization of SETD1A haploinsufficiency in humans and Drosophila defines a novel neurodevelopmental syndrome. Mol. Psychiatry.

[23] Zhou X., Long J.M., Geyer M.A., Masliah E., Kelsoe J.R., Wynshaw-Boris A., Chien K.R. (2005). Reduced expression of the Sp4 gene in mice causes deficits in sensorimotor gating and memory associated with hippocampal vacuolization. Mol. Psychiatry.

[24] Ha C., Lim K. (2015). O-GlcNAc modification of Sp3 and Sp4 transcription factors negatively regulates their transcriptional activities. Biochem. Biophys. Res. Commun..

[25] Priya A., Johar K., Nair B., Wong-Riley M.T. (2014). Specificity protein 4 (Sp4) regulates the transcription of AMPA receptor subunit GluA2 (Gria2). Biochim. Biophys. Acta.

[26] Priya A., Johar K., Wong-Riley M.T.T. (2013). Specificity protein 4 functionally regulates the transcription of NMDA receptor subunits GluN1, GluN2A, and GluN2B. Biochim. Biophys. Acta.

[27] Zhang H., Lu J., Wu S. (2020). Sp4 controls constitutive expression of neuronal serine racemase and NF-E2-related factor-2 mediates its induction by valproic acid. Biochim. Biophys. Acta Gene Regul. Mech..

[28] Ishimaru N., Tabuchi A., Hara D., Hayashi H., Sugimoto T., Yasuhara M., Shiota J., Tsuda M. (2007). Regulation of neurotrophin-3 gene transcription by Sp3 and Sp4 in neurons. J. Neurochem..

[29] Zhou X. (2022). Over-representation of potential SP4 target genes within schizophrenia-risk genes. Mol. Psychiatry.

[30] Zhou X., Tang W., Greenwood T.A., Guo S., He L., Geyer M.A., Kelsoe J.R. (2009). Transcription factor SP4 is a susceptibility gene for bipolar disorder. PLoS ONE.

[31] Pinacho R., Villalmanzo N., Lalonde J., Haro J.M., Meana J.J., Gill G., Ramos B. (2011). The transcription factor SP4 is reduced in postmortem cerebellum of bipolar disorder subjects: Control by depolarization and lithium. Bipolar Disord..

[32] Hara T., Takamura A., Kishi C., Iemura S., Natsume T., Guan J.L., Mizushima N. (2008). FIP200, a ULK-interacting protein, is required for autophagosome formation in mammalian cells. J. Cell Biol..

[33] Wang C., Liang C.C., Bian Z.C., Zhu Y., Guan J.L. (2013). FIP200 is required for maintenance and differentiation of postnatal neural stem cells. Nat. Neurosci..

[34] Chano T., Okabe H., Hulette C.M. (2007). RB1CC1 insufficiency causes neuronal atrophy through mTOR signaling alteration and involved in the pathology of Alzheimer’s diseases. Brain Res..

[35] Wen J., Zellner A., Braun N.C., Bajaj T., Gassen N.C., Peitz M., Brustle O. (2023). Loss of function of FIP200 in human pluripotent stem cell-derived neurons leads to axonal pathology and hyperactivity. Transl. Psychiatry.

[36] Degenhardt F., Priebe L., Meier S., Lennertz L., Streit F., Witt S.H., Hofmann A., Becker T., Mossner R., Maier W. (2013). Duplications in RB1CC1 are associated with schizophrenia; identification in large European sample sets. Transl. Psychiatry.

[37] Aksu M., Pleiner T., Karaca S., Kappert C., Dehne H.J., Seibel K., Urlaub H., Bohnsack M.T., Gorlich D. (2018). Xpo7 is a broad-spectrum exportin and a nuclear import receptor. J. Cell Biol..

[38] Thompson L.L., Rutherford K.A., Lepage C.C., McManus K.J. (2021). The SCF Complex Is Essential to Maintain Genome and Chromosome Stability. Int. J. Mol. Sci..

[39] Dealy M.J., Nguyen K.V., Lo J., Gstaiger M., Krek W., Elson D., Arbeit J., Kipreos E.T., Johnson R.S. (1999). Loss of Cul1 results in early embryonic lethality and dysregulation of cyclin E. Nat. Genet..

[40] Staropoli J.F., Abeliovich A. (2005). The ubiquitin-proteasome pathway is necessary for maintenance of the postmitotic status of neurons. J. Mol. Neurosci..

[41] Wong J.J., Li S., Lim E.K., Wang Y., Wang C., Zhang H., Kirilly D., Wu C., Liou Y.C., Wang H. (2013). A Cullin1-based SCF E3 ubiquitin ligase targets the InR/PI3K/TOR pathway to regulate neuronal pruning. PLoS Biol..

[42] Boix-Perales H., Horan I., Wise H., Lin H.R., Chuang L.C., Yew P.R., Philpott A. (2007). The E3 ubiquitin ligase skp2 regulates neural differentiation independent from the cell cycle. Neural Dev..

[43] Liao E.H., Hung W., Abrams B., Zhen M. (2004). An SCF-like ubiquitin ligase complex that controls presynaptic differentiation. Nature.

[44] Vadhvani M., Schwedhelm-Domeyer N., Mukherjee C., Stegmuller J. (2013). The centrosomal E3 ubiquitin ligase FBXO31-SCF regulates neuronal morphogenesis and migration. PLoS ONE.

[45] Aggarwal S., Bhowmik A.D., Ramprasad V.L., Murugan S., Dalal A. (2016). A splice site mutation in HERC1 leads to syndromic intellectual disability with macrocephaly and facial dysmorphism: Further delineation of the phenotypic spectrum. Am. J. Med. Genet. A.

[46] Nguyen L.S., Schneider T., Rio M., Moutton S., Siquier-Pernet K., Verny F., Boddaert N., Desguerre I., Munich A., Rosa J.L. (2016). A nonsense variant in HERC1 is associated with intellectual disability, megalencephaly, thick corpus callosum and cerebellar atrophy. Eur. J. Hum. Genet..

[47] Utine G.E., Taskiran E.Z., Kosukcu C., Karaosmanoglu B., Guleray N., Dogan O.A., Kiper P.O., Boduroglu K., Alikasifoglu M. (2017). HERC1 mutations in idiopathic intellectual disability. Eur. J. Med. Genet..

[48] Wassef M., Sotelo C., Cholley B., Brehier A., Thomasset M. (1987). Cerebellar mutations affecting the postnatal survival of Purkinje cells in the mouse disclose a longitudinal pattern of differentially sensitive cells. Dev. Biol..

[49] Mashimo T., Hadjebi O., Amair-Pinedo F., Tsurumi T., Langa F., Serikawa T., Sotelo C., Guenet J.L., Rosa J.L. (2009). Progressive Purkinje cell degeneration in tambaleante mutant mice is a consequence of a missense mutation in HERC1 E3 ubiquitin ligase. PLoS Genet..

[50] Montes-Fernandez M.A., Perez-Villegas E.M., Garcia-Gonzalo F.R., Pedrazza L., Rosa J.L., de Toledo G.A., Armengol J.A. (2020). The HERC1 ubiquitin ligase regulates presynaptic membrane dynamics of central synapses. Sci. Rep..

[51] Perez-Villegas E.M., Negrete-Diaz J.V., Porras-Garcia M.E., Ruiz R., Carrion A.M., Rodriguez-Moreno A., Armengol J.A. (2018). Mutation of the HERC 1 Ubiquitin Ligase Impairs Associative Learning in the Lateral Amygdala. Mol. Neurobiol..

[52] Perez-Villegas E.M., Perez-Rodriguez M., Negrete-Diaz J.V., Ruiz R., Rosa J.L., de Toledo G.A., Rodriguez-Moreno A., Armengol J.A. (2020). HERC1 Ubiquitin Ligase Is Required for Hippocampal Learning and Memory. Front. Neuroanat..

[53] Leresche N., Lambert R.C. (2017). T-type calcium channels in synaptic plasticity. Channels.

[54] Perez-Reyes E. (2003). Molecular physiology of low-voltage-activated t-type calcium channels. Physiol. Rev..

[55] Berecki G., Helbig K.L., Ware T.L., Grinton B., Skraban C.M., Marsh E.D., Berkovic S.F., Petrou S. (2020). Novel Missense CACNA1G Mutations Associated with Infantile-Onset Developmental and Epileptic Encephalopathy. Int. J. Mol. Sci..

[56] Kim J.-i., Miura Y., Li M.-Y., Revah O., Selvaraj S., Birey F., Meng X., Thete M.V., Pavlov S.D., Andersen J. (2023). Human assembloids reveal the consequences of *CACNA1G* gene variants in the thalamocortical pathway. bioRxiv.

[57] Barresi S., Dentici M.L., Manzoni F., Bellacchio E., Agolini E., Pizzi S., Ciolfi A., Tarnopolsky M., Brady L., Garone G. (2020). Infantile-Onset Syndromic Cerebellar Ataxia and CACNA1G Mutations. Pediatr. Neurol..

[58] Singh B., Monteil A., Bidaud I., Sugimoto Y., Suzuki T., Hamano S.i., Oguni H., Osawa M., Alonso M.E., Delgado-Escueta A.V. (2007). Mutational analysis of CACNA1G in idiopathic generalized epilepsy. Hum. Mutat..

[59] Gao W.-J., Snyder M.A. (2013). NMDA hypofunction as a convergence point for progression and symptoms of schizophrenia. Front. Cell. Neurosci..

[60] Liu X.-B., Murray K.D., Jones E.G. (2004). Switching of NMDA Receptor 2A and 2B Subunits at Thalamic and Cortical Synapses during Early Postnatal Development. J. Neurosci..

[61] Gao W.J., Yang S.S., Mack N.R., Chamberlin L.A. (2022). Aberrant maturation and connectivity of prefrontal cortex in schizophrenia-contribution of NMDA receptor development and hypofunction. Mol. Psychiatry.

[62] Elmasri M., Hunter D.W., Winchester G., Bates E.E., Aziz W., Van Der Does D.M., Karachaliou E., Sakimura K., Penn A.C. (2022). Common synaptic phenotypes arising from diverse mutations in the human NMDA receptor subunit GluN2A. Commun. Biol..

[63] Ruzicka W.B., Mohammadi S., Fullard J.F., Davila-Velderrain J., Subburaju S., Tso D.R., Hourihan M., Jiang S., Lee H.C., Bendl J. (2024). Single-cell multi-cohort dissection of the schizophrenia transcriptome. Science.

[64] Harrison P.J., Bannerman D.M. (2023). GRIN2A (NR2A): A gene contributing to glutamatergic involvement in schizophrenia. Mol. Psychiatry.

[65] Strehlow V., Heyne H.O., Vlaskamp D.R.M., Marwick K.F.M., Rudolf G., de Bellescize J., Biskup S., Brilstra E.H., Brouwer O.F., Callenbach P.M.C. (2019). GRIN2A-related disorders: Genotype and functional consequence predict phenotype. Brain.

[66] Wu Y., Arai A.C., Rumbaugh G., Srivastava A.K., Turner G., Hayashi T., Suzuki E., Jiang Y., Zhang L., Rodriguez J. (2007). Mutations in ionotropic AMPA receptor 3 alter channel properties and are associated with moderate cognitive impairment in humans. Proc. Natl. Acad. Sci. USA.

[67] Philips A.K., Siren A., Avela K., Somer M., Peippo M., Ahvenainen M., Doagu F., Arvio M., Kaariainen H., Van Esch H. (2014). X-exome sequencing in Finnish families with intellectual disability--four novel mutations and two novel syndromic phenotypes. Orphanet J. Rare Dis..

[68] Greger I.H., Ziff E.B., Penn A.C. (2007). Molecular determinants of AMPA receptor subunit assembly. Trends Neurosci..

[69] Italia M., Ferrari E., Di Luca M., Gardoni F. (2021). GluA3-containing AMPA receptors: From physiology to synaptic dysfunction in brain disorders. Neurobiol. Dis..

[70] Renner M.C., Albers E.H., Gutierrez-Castellanos N., Reinders N.R., van Huijstee A.N., Xiong H., Lodder T.R., Kessels H.W. (2017). Synaptic plasticity through activation of GluA3-containing AMPA-receptors. Elife.

[71] Davies B., Brown L.A., Cais O., Watson J., Clayton A.J., Chang V.T., Biggs D., Preece C., Hernandez-Pliego P., Krohn J. (2017). A point mutation in the ion conduction pore of AMPA receptor GRIA3 causes dramatically perturbed sleep patterns as well as intellectual disability. Hum. Mol. Genet..

[72] Adamczyk A., Mejias R., Takamiya K., Yocum J., Krasnova I.N., Calderon J., Cadet J.L., Huganir R.L., Pletnikov M.V., Wang T. (2012). GluA3-deficiency in mice is associated with increased social and aggressive behavior and elevated dopamine in striatum. Behav. Brain Res..

[73] Peng S.X., Pei J., Rinaldi B., Chen J., Ge Y.H., Jia M., Wang J., Delahaye-Duriez A., Sun J.H., Zang Y.Y. (2022). Dysfunction of AMPA receptor GluA3 is associated with aggressive behavior in human. Mol. Psychiatry.

[74] Cho W., Shin W.S., An I., Bang M., Cho D.Y., Lee S.H. (2019). Biological Aspects of Aggression and Violence in Schizophrenia. Clin. Psychopharmacol. Neurosci..

[75] Rinaldi B., Bayat A., Zachariassen L.G., Sun J.H., Ge Y.H., Zhao D., Bonde K., Madsen L.H., Awad I.A.A., Bagiran D. (2024). Gain-of-function and loss-of-function variants in GRIA3 lead to distinct neurodevelopmental phenotypes. Brain.

[76] Howes O.D., Kapur S. (2009). The dopamine hypothesis of schizophrenia: Version III--the final common pathway. Schizophr. Bull..

[77] Weinberger D.R. (1987). Implications of normal brain development for the pathogenesis of schizophrenia. Arch. Gen. Psychiatry.

[78] Murray R.M., Bhavsar V., Tripoli G., Howes O. (2017). 30 Years on: How the Neurodevelopmental Hypothesis of Schizophrenia Morphed Into the Developmental Risk Factor Model of Psychosis. Schizophr. Bull..

[79] Weinberger D.R. (1996). On the plausibility of “the neurodevelopmental hypothesis” of schizophrenia. Neuropsychopharmacology.

[80] Weinberger D.R. (2017). Future of Days Past: Neurodevelopment and Schizophrenia. Schizophr. Bull..

[81] Keshavan M.S., Hogarty G.E. (1999). Brain maturational processes and delayed onset in schizophrenia. Dev. Psychopathol..

[82] van der Meer D., Cheng W., Rokicki J., Fernandez-Cabello S., Shadrin A., Smeland O.B., Ehrhart F., Gülöksüz S., Pries L.-K., Lin B. (2023). Clustering Schizophrenia Genes by Their Temporal Expression Patterns Aids Functional Interpretation. Schizophr. Bull..

[83] Miller, J.A., Ding S.L., Sunkin S.M., Ng L., Szafer A., Ebbert A., Riley Z.L., Royall J.J., Aiona K., Arnold J.M. (2024). Transcriptional landscape of the prenatal human brain. Nature.

[84] Clarin J.D., Reddy N., Alexandropoulos C., Gao W.J. (2024). The role of cell adhesion molecule IgSF9b at the inhibitory synapse and psychiatric disease. Neurosci. Biobehav. Rev..

[85] Bilecki W., Mackowiak M. (2023). Gene Expression and Epigenetic Regulation in the Prefrontal Cortex of Schizophrenia. Genes.

[86] Skene N.G., Roy M., Grant S.G. (2017). A genomic lifespan program that reorganises the young adult brain is targeted in schizophrenia. Elife.

[87] Munguba H., Chattopadhyaya B., Nilsson S., Carrico J.N., Memic F., Oberst P., Batista-Brito R., Munoz-Manchado A.B., Wegner M., Fishell G. (2021). Postnatal Sox6 Regulates Synaptic Function of Cortical Parvalbumin-Expressing Neurons. J. Neurosci..

[88] Batista-Brito R., Rossignol E., Hjerling-Leffler J., Denaxa M., Wegner M., Lefebvre V., Pachnis V., Fishell G. (2009). The cell-intrinsic requirement of Sox6 for cortical interneuron development. Neuron.

[89] McHugo M., Talati P., Armstrong K., Vandekar S.N., Blackford J.U., Woodward N.D., Heckers S. (2019). Hyperactivity and Reduced Activation of Anterior Hippocampus in Early Psychosis. Am. J. Psychiatry.

[90] Pinkham A.E., Liu P., Lu H., Kriegsman M., Simpson C., Tamminga C. (2015). Amygdala Hyperactivity at Rest in Paranoid Individuals With Schizophrenia. Am. J. Psychiatry.

[91] Dienel S.J., Lewis D.A. (2019). Alterations in cortical interneurons and cognitive function in schizophrenia. Neurobiol. Dis..

[92] Glausier J.R., Lewis D.A. (2013). Dendritic spine pathology in schizophrenia. Neuroscience.

[93] Guo J.Y., Ragland J.D., Carter C.S. (2019). Memory and cognition in schizophrenia. Mol. Psychiatry.

[94] Heckers S., Rauch S.L., Goff D., Savage C.R., Schacter D.L., Fischman A.J., Alpert N.M. (1998). Impaired recruitment of the hippocampus during conscious recollection in schizophrenia. Nat. Neurosci..

[95] Pirnia T., Woods R.P., Hamilton L.S., Lyden H., Joshi S.H., Asarnow R.F., Nuechterlein K.H., Narr K.L. (2015). Hippocampal dysfunction during declarative memory encoding in schizophrenia and effects of genetic liability. Schizophr. Res..

[96] Birnbaum R., Weinberger D.R. (2017). Genetic insights into the neurodevelopmental origins of schizophrenia. Nat. Rev. Neurosci..

[97] Clementz B.A., Sweeney J.A., Hamm J.P., Ivleva E.I., Ethridge L.E., Pearlson G.D., Keshavan M.S., Tamminga C.A. (2016). Identification of Distinct Psychosis Biotypes Using Brain-Based Biomarkers. Am. J. Psychiatry.

[98] Brown A.S. (2011). The environment and susceptibility to schizophrenia. Prog. Neurobiol..

[99] Robinson N., Bergen S.E. (2021). Environmental Risk Factors for Schizophrenia and Bipolar Disorder and Their Relationship to Genetic Risk: Current Knowledge and Future Directions. Front. Genet..

[100] Focking M., Doyle B., Munawar N., Dillon E.T., Cotter D., Cagney G. (2019). Epigenetic Factors in Schizophrenia: Mechanisms and Experimental Approaches. Mol. Neuropsychiatry.

[101] Li R., Ma X., Wang G., Yang J., Wang C. (2016). Why sex differences in schizophrenia?. J. Transl. Neurosci..

[102] Li X., Zhou W., Yi Z. (2022). A glimpse of gender differences in schizophrenia. Gen. Psychiatr..

[103] International Schizophrenia C., Purcell S.M., Wray N.R., Stone J.L., Visscher P.M., O’Donovan M.C., Sullivan P.F., Sklar P. (2009). Common polygenic variation contributes to risk of schizophrenia and bipolar disorder. Nature.

[104] Vassos E., Di Forti M., Coleman J., Iyegbe C., Prata D., Euesden J., O’Reilly P., Curtis C., Kolliakou A., Patel H. (2017). An Examination of Polygenic Score Risk Prediction in Individuals With First-Episode Psychosis. Biol. Psychiatry.

[105] Perkins D.O., Olde Loohuis L., Barbee J., Ford J., Jeffries C.D., Addington J., Bearden C.E., Cadenhead K.S., Cannon T.D., Cornblatt B.A. (2020). Polygenic Risk Score Contribution to Psychosis Prediction in a Target Population of Persons at Clinical High Risk. Am. J. Psychiatry.

[106] Palk A.C., Dalvie S., de Vries J., Martin A.R., Stein D.J. (2019). Potential use of clinical polygenic risk scores in psychiatry-ethical implications and communicating high polygenic risk. Philos. Ethics Humanit. Med..

[107] Ryan J., Virani A., Austin J.C. (2015). Ethical issues associated with genetic counseling in the context of adolescent psychiatry. Appl. Transl. Genom..

[108] Dataset: Allen Institute for Brain Science. 2010. Allen Developing Human Brain Atlas: Developmental Transcriptome [Dataset]. RRID:SCR_008083. https://brainspan.org.

